# Seeding of a Pituitary Adenoma or Atypical Pituitary Carcinoma?

**DOI:** 10.7759/cureus.1211

**Published:** 2017-05-02

**Authors:** Evan M Krueger, Jason Seibly

**Affiliations:** 1 Neurosurgery, Advocate Health Care; 2 Neurosurgery, Central Illinois

**Keywords:** pituitary adenoma, pituitary carcinoma, recurrence

## Abstract

Pituitary carcinomas are defined as pituitary tumors with craniospinal and/or systemic metastasis. These are rare and highly aggressive lesions. We present an unusual case of a 52-year-old male who had a pituitary adenoma removed via craniotomy. The tumor recurred three years post-op near the surgical tract, and slowly enlarged before removal two years later. Technically, this lesion was defined as a pituitary carcinoma, even though the histology and clinical course were atypical. There is no standardized grading system for pituitary tumors and ideal criteria should correlate clinically. Treatment for pituitary carcinoma is multimodal and largely empiric. We believe this case illustrates that current definitions of pituitary carcinoma are incomplete.

## Introduction

Pituitary carcinomas are defined as pituitary tumors with craniospinal and/or systemic metastasis [1]. These variants represent only 0.2% of all pituitary tumors [2]. Pituitary carcinomas are thought to arise de novo, or more frequently from malignant transformation of an adenoma [3]. The mean latency period between initial presentation and transformation is 6.6 years [2]. The vast majority of carcinomas are functional [2]. These are highly aggressive lesions, with historically 66% of patients dying within one year of diagnosis [2].

Pituitary adenomas, by contrast, are more common lesions that are typically benign. However, in a large case series, recurrence rates of 30% for adenomas after a transcranial approach have been reported [4]. We present a rare case of either seeding of a pituitary adenoma or an atypical pituitary carcinoma. This illustrates the need for improved clinical definitions to guide treatment.

## Case presentation

**Figure 1:**
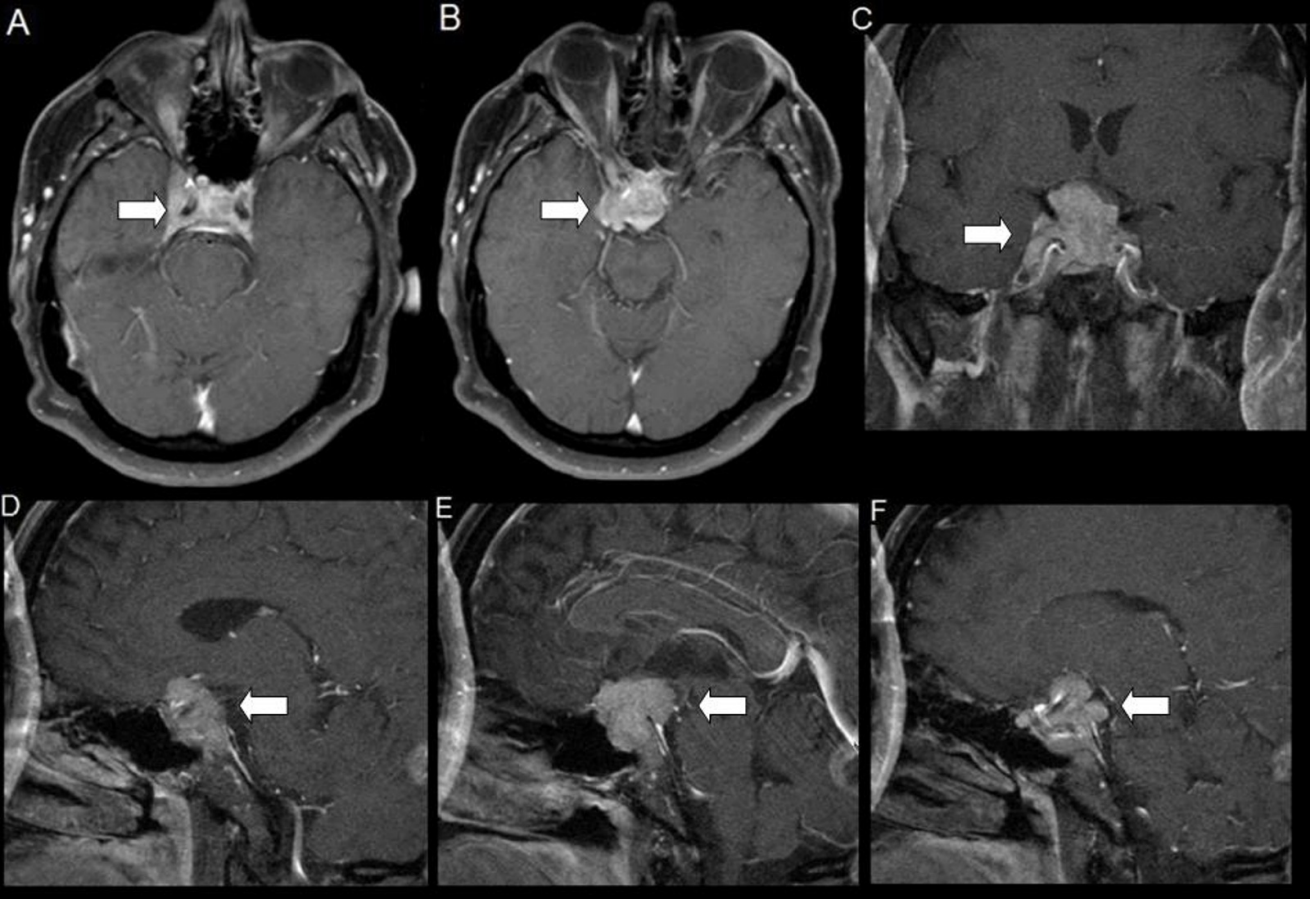
Contrast Enhanced Brain Magnetic Resonance Imaging (MRI) on Presentation Axial (A, B), Coronal (C), and Sagittal (D, E, and F) images showed an enhancing, lobulated mass in the sella arising from the pituitary enveloping the right carotid siphon, right cavernous sinus, optic chiasm, and right optic nerve. The parenchyma was otherwise normal.

A 52-year-old male presented with a chief complaint of insidious onset of right monocular vision loss. There was no significant past medical, surgical, familial, or social history. Endocrine symptomatology and workup were unremarkable. A contrast-enhanced brain magnetic resonance imaging (MRI) revealed an infiltrating, enhancing mass arising from the pituitary (Figure 1).

**Figure 2:**
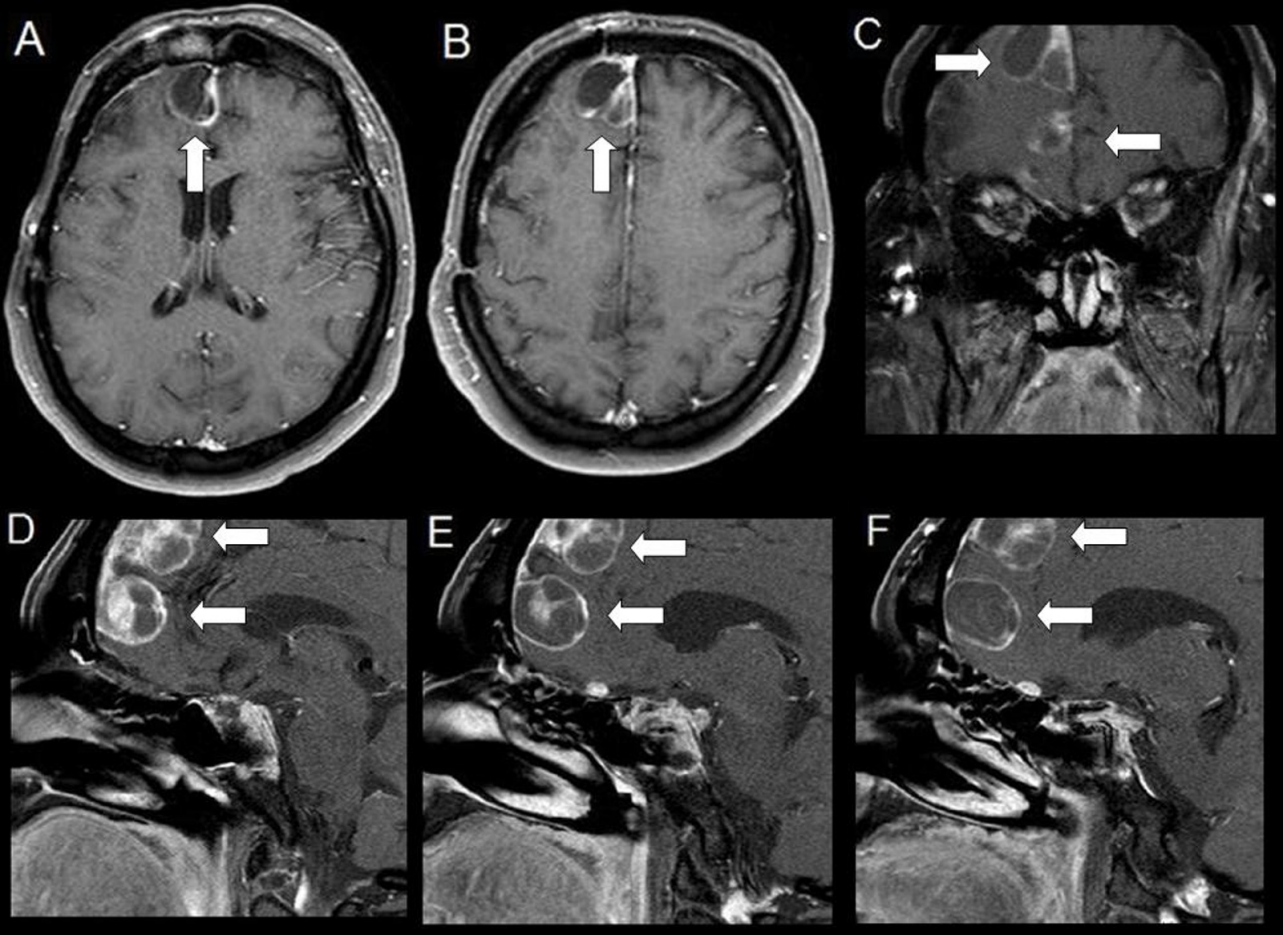
Contrast Enhanced Brain Magnetic Resonance Imaging (MRI) Two Years After Discovery of the Right Frontal Lesions, and Five Years After Initial Craniotomy Axial (A, B), Coronal (C), and Sagittal (D, E, and F) images showed peripherally enhancing, cystic structures along the anterior parafalcine and cribriform regions of the right front lobe. There was stable, slight residual enhancement of the sella turcica, and right cavernous sinus and carotid artery. The parenchyma was otherwise normal.

He underwent a right orbital-zygomatic osteotomy with a Dolenc procedure. Pathology revealed a silent corticotroph adenoma with irregular cytomorphology and architecture, five mitosis per 10 high-power fields, and only mildly evaluated MIB-1 labeling and p53 protein. Three months post-operatively he underwent CyberKnife radiotherapy (25 Gy divided into five fractions) for residual tumor encasing the right cavernous sinus and carotid artery. Surveillance imaging for 2.5 years post-operatively was unremarkable. However, three years post-operatively, he returned with altered mental status. A contrast-enhanced brain MRI showed new enhancing extra-axial masses along the right anterior interhemispheric falx measuring 14 × 7 mm and 9 × 7 mm. Cerebrospinal fluid (CSF) samples showed bacterial meningitis and negative cytology. A cisternogram confirmed a CSF leak that was repaired transsphenoidally. The new extra-axial lesions were initially concerning for cerebral abscesses, and were treated with long-term intravenous antibiotics. He improved and remained asymptomatic for the next two years. Incidentally, he underwent an elective cervical fusion for spondylosis during this interim and cervical MRI did not show evidence of systemic disease. The patient was reluctant to undergo additional cranial procedures, however, the interhemispheric lesions gradually enlarged to 23 × 20 mm and 21 × 16 mm (Figure 2).

**Figure 3:**
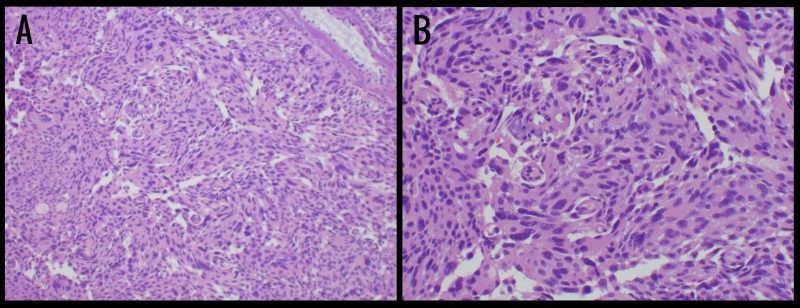
Histology of Right Frontal Lesions After Second Craniotomy Hematoxylin and eosin stained sections (A and B) showed large atypical hyperchromatic nuclei, prominent nucleoli, moderate to ample amphophilic cytoplasm, and considerable polymorphism. Specimen also had 5 mitosis per 10 high power field, only mildly elevated MIB-1 labeling and p53 protein, and immunohistochemical staining positive for adrenocorticotropic hormone (ACTH) but negative for all other pituitary hormones.

Therefore, the patient underwent a right frontal craniotomy for tissue diagnosis and gross total resection. The tumor was well encapsulated but not adherent to the surrounding parenchyma. Pathology once again showed an atypical corticotroph adenomas, unchanged since five years prior (Figure 3).

We cannot definitively state if these lesions seeded along the surgical tract or represent an organic carcinoma. Thus, in our opinion, the patient warranted careful clinical and radiographic observation. Further recurrence would prompt additional consideration for radiation and or chemotherapy.

## Discussion

The origins of the right frontal masses in this case are speculative. Spontaneous ectopic adenomas seem unlikely given similar histology as initial presentation. While our patient received CyberKnife therapy, there is no evidence to support radiation-induced pituitary adenomas specifically [5]. Another explanation in our case is iatrogenic seeding along the surgical tract [6]. Cells from an acapsular tumor could spread in the subarachnoid space via inherent rupture of Liliequist's Membrane and become arrested at the arachnoid granulations, or via valveless anastomotic cerebral veins such as Trolard. Perhaps we removed the lesion as a carcinoma early in its course, which raises questions about follow-up intervals. It is unknown if potential surgical approach specific recurrence risks outweigh resection goals, or have surveillance implications.

The clinical course in this case is highly unusual. Unlike typical pituitary carcinomas, there was shorter latency period, the primary and secondary lesions were clinically non-functioning, and the patient has had a longer progression-free survival. Others have proposed dissemination of an adenoma does not equate to malignancy [7]. Furthermore, other cases have shown benign histology on lesions technically defined as carcinoma due to intra-cranial metastasis [6]. Our patient may have an atypical course for pituitary carcinoma, or illustrate that current disease definitions are not always applicable clinically.

There is not unified pituitary tumor nomenclature. Classically, these lesions were described by their size, staining, and functional status. In 2004, the World Health Organization (WHO) proposed a new system which included typical and atypical adenomas, and pituitary carcinoma [1]. Atypical tumors were thought to have uncertain behavior, and were defined as invasive growth, Ki-67 labeling index greater than three percent, 'elevated' mitotic activity, and 'extensive' nuclear staining for p53 [1]. This has been confirmed and specified showing p53 ≥ 2% and mitotic index ≥2 within 10 high power fields are appropriate thresholds [8]. To further distinguish between atypical adenomas and pituitary carcinoma, criteria such as invasion, neoangiogenesis, vascular invasion, abnormal mitosis, markedly high Ki-67 and p53, and/or genomic alteration have been postulated, but not well described [7-8]. Interestingly, one study reported no differences between atypical adenomas and pituitary carcinoma in regard to Ki-67, p53, mitotic index, and invasiveness [8]. A promising classification scheme combing histologic and radiographic criteria seems to predict disease-free and recurrence/progression-free status, but will require validation [9]. Ultimately, no widely accepted genetic, histologic, or biomarkers have been identified that predict disease progression [3, 10]. Ideally, a unified grading and staging system would predict behavior to identify patients who may benefit from early intervention to prevent disease progression.

Treatment for pituitary carcinomas typically includes some combination of surgery, radiotherapy, adjuvant medical therapy, peptide receptor radionuclide therapy, and/or chemotherapy [3, 10]. Recently, Temozolomide has received greater interest with aggregate carcinoma data from four cohort studies showing 55.5% and 72% response and stabilization rates, respectively [10]. Chatzellis, et al. provide a treatment algorithm for aggressive lesions based on clinical experience [10]. Future directions include defining the candidates, initiation, and goals of these therapies [3, 10].

## Conclusions

In conclusion, we present an unusual case of either a pituitary adenoma that seeded into the right frontal lobe after initial craniotomy or an atypical pituitary carcinoma. In our opinion, it seems insufficient to diagnosis pituitary carcinoma solely based on distant lesions. Tissue research has provided criteria that do not yet guide clinical decisions. For cases of probable malignancy, treatment largely remains empiric. This case may be used in aggregate to improve future clinically relevant disease definitions.
